# Developing a Sustainable and Circular Bio-Based Economy in EU: By Partnering Across Sectors, Upscaling and Using New Knowledge Faster, and For the Benefit of Climate, Environment & Biodiversity, and People & Business

**DOI:** 10.3389/fbioe.2020.619066

**Published:** 2021-01-21

**Authors:** Lene Lange, Kevin O. Connor, Sigurjon Arason, Uffe Bundgård-Jørgensen, Antonella Canalis, Dirk Carrez, Joe Gallagher, Niels Gøtke, Christian Huyghe, Bruno Jarry, Pilar Llorente, Mariya Marinova, Ligia O. Martins, Philippe Mengal, Paola Paiano, Calliope Panoutsou, Ligia Rodrigues, Dagmar B. Stengel, Yvonne van der Meer, Helena Vieira

**Affiliations:** ^1^BioEconomy, Research and Advisory, Copenhagen, Denmark; ^2^BiOrbic SFI Bioeconomy Research Centre, School of Biomolecular and Biomedical Science, University College Dublin, Dublin, Ireland; ^3^Faculty of Food Science and Nutrition in the University of Iceland and Matis ohf, Reykjavík, Iceland; ^4^Gate2Growth, DTU Science Park, Kongens Lyngby, Denmark; ^5^Bio-based Industries Joint Undertaking (BBI JU), Brussels, Belgium; ^6^Bio-based Industries Consortium (BIC), Brussels, Belgium; ^7^Institute of Biological, Environmental and Rural sciences (IBERS), Aberystwyth University, Aberystwyth, United Kingdom; ^8^Ministry of Science, Innovation and Higher Education of Denmark, Copenhagen, Denmark; ^9^Scientific Director Agriculture, INRAE, Paris, France; ^10^National Academy of Technology of France, Paris, France; ^11^Department of Chemistry and Chemical Engineering, Royal Military College of Canada, Kingston, ON, Canada; ^12^Instituto de Tecnologia Química e Biológica, Universidade Nova de Lisboa, Oeiras, Portugal; ^13^Centre for Environmental Policy, Imperial College London, London, United Kingdom; ^14^Centre of Biological Engineering, University of Minho, Campus de Gualtar, Braga, Portugal; ^15^Botany and Plant Science, School of Natural Sciences, National University of Ireland Galway, Galway, Ireland; ^16^Ryan Institute, National University of Ireland Galway, Galway, Ireland; ^17^Aachen Maastricht Institute for Biobased Materials, Maastricht University, Brightlands Chemelot Campus, Geleen, Netherlands; ^18^Directorate General of Maritime Policy, Ministry of the Sea, and BioISI – Biosystems & Integrative Sciences Institute, Faculty of Sciences, University of Lisbon, Lisbon, Portugal

**Keywords:** biorefinery technologies, bio-based products, microbial production, upgrading, side-streams & wastes, Bio-Based Industries Joint Undertaking (BBI-JU), Biobased Industries Consortium (BIC), Circular Bio-based Economy (CBE)

## Abstract

This paper gives an overview of development of the EU-bioeconomy, 2014–2020. The Vision of the new Circular Bio-based Economy, CBE is presented: Unlocking the full potential of all types of sustainably sourced biomass, crop residues, industrial side-streams, and wastes by transforming it into value-added products. The resulting product portfolio consists of a wide spectrum of value-added products, addressing societal and consumer needs. Food and feed, bio-based chemicals, materials, health-promoting products; and bio-based fuels. The pillars of CBE are described, including biotechnology, microbial production, enzyme technology, green chemistry, integrated physical/chemical processing, policies, conducive framework conditions and public private partnerships. Drivers of CBE are analyzed: Biomass supply, biorefineries, value chain clusters, rural development, farmers, foresters and mariners; urgent need for climate change mitigation and adaptation, and stopping biodiversity loss. Improved framework conditions can be drivers but also obstacles if not updated to the era of circularity. Key figures, across the entire BBI-JU project portfolio (2014–2020) are provided, including expansion into biomass feedstocks, terrestrial and aquatic, and an impressive broadening of bio-based product portfolio, including higher-value, health-promoting products for man, animal, plants and soil. Parallel to this, diversification of industrial segments and types of funding instruments developed, reflecting industrial needs and academic research involvement. Impact assessment is highlighted. A number of specific recommendations are given; e.g., including international win/win CBE-collaborations, as e.g., expanding African EU collaboration into CBE. In contrast to fossil resources biological resources are found worldwide. In its outset, circular bio-based economy, can be implemented all over, in a just manner, not the least stimulating rural development.

## Introduction

The new bioeconomy vision is to unlock the full potential of all types of sustainably sourced biomass including residual biomasses, such as crop residues, industrial side-streams, and food waste, (estimated by FAO worldwide to be one third of the product produced) (FAO, 2019), as well as organic municipality waste by transforming it into value-added products. Biorefinery technologies provide a route to improved use of the biological resources. The new bioeconomy also includes upgraded valorization of hitherto discarded/downgraded parts of the plants and animals including aquatic (fresh water and marine) resources (BBI-JU Macrocascade) (Macrocascade, 2018). Notably, the resulting bio-based product portfolio consists of a wide spectrum of value-added products, addressing a series of societal and consumer needs. Not just more food and feed, but also bio-based chemicals, materials, health-promoting products (such as drugs, skin- and wound-care and gut-health promoting food and feed ingredients) and bio-based fuels. The new bioeconomy is referred to as the “bio-based economy”, to distinguish it from the classical bioeconomy, consisting of agricultural-derived products (cereals, vegetables and meat), and conventional products from fishery and forestry. However, the updated EU Bioeconomy strategy in 2018 (European Commission, 2018), the EU Green deal in 2020 (European Commission, 2019c) and the Circular Economy action Plan in 2020 (European Commission, 2020a), are clearly integrating food and non-food production as part of a strategy to develop a single coherent bio-based economy (Philippidis et al., 2018a).

This paper gives a brief introduction to the history and development of the modern bioeconomy in Europe. It includes drivers that led to the development of the first EU Bioeconomy strategy (in 2012, European Commission, 2012), the EU Strategic Innovation and Research Agenda (SIRA, 2013, BIC, 2013) and of policies relevant for the bioeconomy. An overview will be given of the major influencers of the revision of the new EU bioeconomy strategy (2018, European Commission, 2018), including the climate change agenda, COP-meetings, Conference of the Parties (Parker and Karlsson, 2018), the 17 UN SDGs (UN SDGs, 2020), EU Green Deal (European Commission, 2019c), EU CAP reform (CAP Reform, 2019), the updated SIRA (2017) (BIC, 2017), KBBE (knowledge-based bioeconomy) (European Commission, 2020e) and Sustainable Chemistry, SusChem) (SusChem, 2018). Such policy developments are underpinning research and progress in the areas of sustainable biomass supply, conversion technologies (including downstream purification), product development, scaling of technologies, emissions reduction, resource-efficiency, and more recently biodiversity, and eco-system services. Such advances are further attempting to reduce the time to market but also to create a structuring effect through increased collaboration and decreased fragmentation in the sector, which will also drive the growth of new business models (Philippidis et al., 2018a).

Central to the structuring effect of developing a true bioeconomy in Europe was the formation of a public private partnership, the Bio-based Industries Joint Undertaking (BBI-JU), which created a new program of research and development. The BBI-JU represents the largest EU investment in bioeconomy research and development (€3.7 Billion over seven years) contributed jointly by public and private investments. This investment funded research projects at low and high technology readiness level (TRL), as well as cross cutting research known as co-ordination and support actions (CSAs).

The evolution of the BBI-JU annual work program from 2014–2020 has been characterized by a diversification of biomass resources to be valorized into a similarly rich diversity of products and the evolution of thinking from traditional to cross cutting value chains and more recently to cross cutting circular supply chains. The new bioeconomy, developed under external pressures, was based on concern for the environment, climate change, biodiversity loss, and awareness of the need for more responsible consumption, while supporting the economy and developing employment for many types of skills. It focused on principles such as sustainability, circularity and inclusivity (Fritsche and Iriarte, 2014); it draws upon a widespread of biomass materials from aquatic to forest, including by-products and side-streams of other industries and even to multimix waste stream bases; and it is focused on developing new kinds of bio-based products to substitute and replace old fossil-based products. It aims at open up new markets and creating new value chains. Entrepreneurial and innovative thinking in the sector, over the last ten years, has significantly diversified the bio-based portfolio including higher value chemicals and materials and not least higher value nutritional products for the benefit of human and animal health. Unlocking the full potential of the biomass became the unifying mantra. Using the bio-based thinking and circular economy principles have led the way to open up new markets and drive new innovation (BBI-JU, 2020c).

The upgrade of side streams and residues from the harvesting and processing of biological resources is critically important as it significantly increases resource efficiency, thereby lowering the carbon footprint for each ton of final product produced, while also improving economic sustainability. The inclusion of food waste, side-streams and byproducts from food processing opens up additional opportunities for society to capture benefits from its resources for the production of value-added bio-based products. This can reduce pressure on land and environment (e.g., fertilizer, pesticides, and water) and leave more space and suitable habitats to biodiversity, contributing to stopping the loss of biodiversity (EU 2020 Biodiversity Strategy) (Europe Central Asia, 2020). Notably, at this point in time, policy development and regulatory schemes will need to catch up with technology developments to allow safe use of wastes as a resource for the production of bio-based products.

The renewability of biomass does not in itself, indicate sustainability. Sustainable supply of biomass, from primary production, agriculture, forestry, fishery or aquaculture, as well as from industrial processing and organic multimix wastes remains a major challenge; a challenge, which needs to be addressed as an integrated part of all current and future bio-based productions. Life cycle analysis, sustainability criteria and 360-degree, holistic thinking are essential and important components of biomass use and technology assessment (Fritsche and Iriarte, 2014). More recently for the latter, such analysis is being brought in at an even earlier stage in technology development. The sustainability criteria must be expanded to also include impact on habitat ecology in general and on biodiversity in particular. Notably, the bio-based economy is seen to have potential for significant contributions to the climate change agenda by unlocking the full potential of all biological resources produced. This also includes new bio-based products and solutions, substituting for fossil-based products, and also bio-actives, as well as non-toxic bio-based alternatives to toxic pesticides used in both agriculture and the aquatic sector. While there will be substitution of fossil-based products, there will also be the use of nature-based solutions, which could, for example, remove or reduce the need for the addition of chemicals in agriculture. Achieving truly sustainable production and responsible consumption, living up to all three criteria of sustainability; environmental, economic and social, needs to be carefully regulated and monitored (University of Wisconsin, 2017).

Fossil resources are globally unevenly distributed. In contrast to this, biological resources are found all over the world. It is to be expected that the idea of multiple geographically delineated bio-economies (Nordic, Mediterranean, Central and Eastern Europe, Asia, North and South America and African) will be developed. For all these regions, exploitation of biological resources can stimulate development of rural and local areas worldwide. Global accessibility of efficient biomass upgrading technologies, and skills and competence will most probably lead to more raw materials being upgraded and valorized in the country, where they are generated. The benefit achieved by local upgrading of raw materials to high value products includes local job creation, and social and economic livelihood. Thus, motivation for local upgrade instead of export of low-priced primary production products are both prominent and strong.

## The Pillars for Building the Bio-Based Economy in Europe

### Biotechnology

#### Microbial Production

Over decades, methods for biological production of valorized products were developed and implemented (drugs, e.g., statins and human insulin; enzymes and organic acids). This progress was built through decades of strong and integrated collaboration between academia and industrial R&D, taking advantage of the global microbial resources and discovery, was well as steep learning curve in understanding the cell’s manufacturing pathways, especially knowing and using DNA, RNA and protein-derived technologies, as well as pathway engineering for smaller molecules. The competitiveness of microbial-based production was driven forward by the market pull for producing molecules for bio-actives or materials that were not easily achieved by chemical synthesis or were less costly to produce using microorganisms as compared to producing it by chemical processing. Similarly, a strong market-pull for microbially produced industrial enzymes evolved, cumulatively opening for more environmentally benign processing as well as improved use of the raw materials. Notably, in the last one to two decades biological production of complex, biologically derived molecules, like antibodies and hormones are in high demand for the benefit of human health (Kesik-Brodacka, 2018).

#### Enzyme Technology and Bio-Based Concepts

The bio-based concept developed alongside the development of advanced enzyme technology: First, microbially derived enzyme products were developed and produced under the umbrella of more environmentally benign processing by industrial biotechnology companies more than 50 years ago. Secondly, in the 1980’s a new enzyme technology era started using genetically modified production hosts (bacteria or fungi). A highly diverse portfolio of such, “one enzyme at a time” products were developed from the end of the 1980’s and up to today. This generation of enzyme products can be described in short as “One protein, One enzyme function, One product”. A very efficient tool for growing industrial biotechnology, where enzyme processing took over from chemical processing in many industries like textile, detergent, forestry and feed and food processing, including baking (Abraham, 2017; Lange et al., 2017).

The third type of enzyme products, for industrial processing was developed around the start of the new millennium to overcome the “recalcitrant biomass conversion challenge” that lignocellulosic materials posed. Eventually, enzymatic degradation of the recalcitrant lignocellulose structure was enabled by producing a carefully designed blend of enzymes, often produced by a single multi-modified production strain with the latter needed to keep costs of enzymes down. The blends of enzymes for biomass conversion provided the basis for a new “Bio-based Era” for a wide range of industries resulting in a huge portfolio of products (bio-based fuels, chemical building blocks, polymers, materials, feed additives, food ingredients, cosmeceuticals, health promoting products and pharmaceuticals) (Lange, 2017).

#### Green Chemistry

Over decades, methods for chemical synthesis and processing were developed. Again, close collaboration between industrial and public research was instrumental for establishing and growing a strong chemical industry in Europe. Since the nineteen eighties, a new Green Chemistry discipline evolved, aiming at using less hazardous chemical syntheses, designing more environmentally benign chemicals, using safer solvents, and optimizing processes with improved energy efficiency (EPA, 2017), EPA defining Green Chemistry. This Green Chemistry trend, within chemical manufacturing, also developed into refining and broadening the use of chemical catalysts to reduce hazardous emissions and to improve process efficiency. Here also, Green Chemistry, commenced a transition away from fossil toward renewable feedstock. This in turn led to technology developments for more efficient product recovery and down-stream processing. This platform of chemical knowledge is a rich toolbox that can be used in an integrated manner to develop efficient bio-based production technologies.

#### Advanced Physical and Chemical Processing

Combining bio-catalysis and chemical catalysis with physical and chemical processing technologies enabled more efficient use of the feedstock and more cost-efficient biorefining procedures. Advanced membrane technologies have enabled efficient product recovery; and know-how within chemical and material science disciplines enabled the development of biorefinery processes and products, with new functionalities, and thus going far beyond simply making drop-in products to substitute fossil-based products (BBI-JU, Nova reporting, BBI-JU, 2018). These coinciding technological developments opened up a vision of a more sustainable, bio-based society, moving away from a fossil-based world and addressing the challenges of climate change.

Policy: Establishing policies and conducive framework conditions

Under the umbrella of the common EU Research funding system (Frame Work Programs 1–7, and Horizon 2020), the KBBE activities (Knowledge Based BioEconomy European Commission, 2020e), as well as the Sustainable Chemistry [SusChem (2018)] took form. On this basis, a dedicated EU Bioeconomy Strategy was developed (European Commission, 2012). DG Grow hosted the lead market initiative for bio-based products, and subsequently the expert group for bio-based products looked at the industry and policy landscape and measures to stimulate the wider establishment of bio-based industries in Europe and the uptake of these products into the market. DG Research hosted Advisory Groups for the Biotechnology, Food and Agricultural area, DG MARE hosted expert groups on Ocean Economy and Marine Biotechnology and also the Standing Committee on Agricultural Research (SCAR) committee (SCAR, 2020). The formation of the Bio-based Industries Consortium (BIC) (BIC, 2019), discussions about a Public Private Partnership (PPP) and the European Bioeconomy Strategy (2012, European Commission, 2012) arose from activities and recommendations of these groups, which eventually led to the formation of the Bio-Based Industries Joint undertaking, BBI-JU (BBI-JU, 2019).

#### Framework: A Public Private Partnership

The BBI-JU Research and Innovation Program had its first call in 2014. It required a partnership between forward thinking industries and public institutions willing to create a shared vision. The progress made under BBI-JU is worth analyzing, learning from and building upon. The BBI-JU provided for the first time a platform for the development and scale-up of biomass conversion technologies from medium to high technology readiness level (TRL 3–8) through the establishment of demonstration projects and flagship biorefineries. While technological advancement and implementation at scale are key drivers of this partnership, the BBI-JU also supports research on policy and market measures for bio-based products. Importantly, BBI-JU was from the beginning designed as an industry-led program, focusing on public private collaboration, using this as a basis for developing technical solutions to overcome obstacles for the advancement of the bio-based industries. As a result, momentum was built for more cost-efficient processing to unlock the full potential of the biomass and the development of higher value products. A major achievement was the fostering of close collaboration between academia and industries; bringing researchers from traditional academic settings to work with industry on research challenges higher up the TRL scale and convincing industry to invest in promising, lower TRL research. This stimulated new knowledge creation, an avenue for more rapid use of new knowledge, new value chains and business opportunities. Importantly, the program fostered SME participation with specific targets for participation and funding. The overall value of the bioconomy in EU can be illustrated by the following few figures: Around €2 trillion in annual turnover; over 18 million people employed in EU Bioeconomy; €621 billion in added value; 4.2% of the EU’s GDP; 76% of employment.

The BBI-JU is informed by a Strategic Innovation and Research Agenda (SIRA) (2013 and 2017) (BIC, 2013, 2017), which set out the major challenges for the bio-based sector in Europe (European Commission, 2020b). Its revision in 2017 reflects an evolving sector and the need to prioritize circularity, resource efficiency, the use of bio-based CO_2_, reduction of GHG emissions and the need to integrate resilience building measures, such as enhancing biodiversity, into central plans for the bioeconomy (EU Biodiversity Strategy, 2020) (Europe Central Asia, 2020).

## The Drivers of the Bioeconomy

**FIGURE 1:**
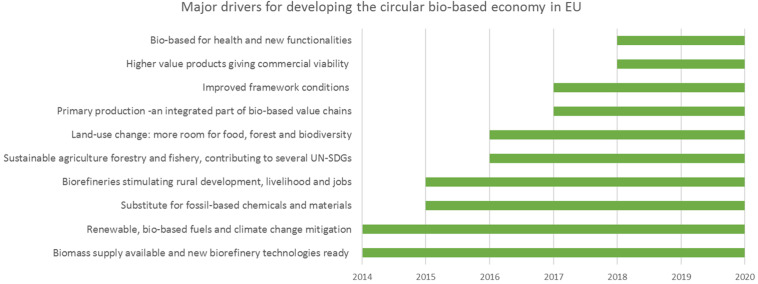
Drivers of developing the EU Circular Biobased Economy.

An overview of major drivers of the EU circular biobased economy, CBE is given in Figure 1.

### Biomass Supply

There is ample feedstock available, but it is also in short supply: Over the entire food chain (crop and fisheries residues, industrial processing side-streams and wastes), FAO estimates that globally we waste approx. one third of all food produced (FAO, 2019). Furthermore, when the loss is a result of not using the newest technologies for opening otherwise recalcitrant plant structures, the total loss in the food chain is estimated to be close to 50%. In the developing world, greater loss is taking place in the field and during storage of primary produce. In industrialized countries, more waste is taking place in the retail and end-user part of the value chain. The resulting loss is, however, almost the same all over the world (FAO, 2019). For the non-food area, significant underexploited side-stream and waste biomass resources are found in all parts of the world, from both primary production and from industrial processing (e.g., cotton/textile or forestry/timber/paper. Limiting feedstock availability, in spite of global overall availability of residues, is still an important obstacle for bio-based industries. Specific feedstock, for which processing technology have been developed, may not be available; the feedstock of interest may have found other uses; or the feedstock planned for (for production of e.g., biofuel) may turn out to be too costly to process into lower value products only, for a robust and competitive business.

### Biorefineries Stimulating Rural Development

Social and societal wellbeing is a strong component of the UN Sustainable Development Goals (SDGs) (UN SDGs, 2020) and of sustainability criteria (Fritsche and Iriarte, 2014; European Commission, 2019b); as such development of biorefineries close to where the crop is being produced is recognized as one of the most important sources for creating new jobs as well as economic and social development in rural areas. This is indeed a strong political and policy driver for strengthening the bio-based economy. Furthermore, this important aspect represents a strong driver for developing also decentralized and smaller scale biorefineries and also end-of-pipe biorefinery solutions installed on existing industrial sites. This is a significant step up and away from the initial phase of the bioeconomy, where the low profit margin gave incentives for very large installments; centrally placed, requiring huge investments and being commercially viable at large scale. The low profit margin of the biomass-to-biofuel biorefinery, caused by the initial focus on biofuels only, a low value product, have been challenging the commercial viability of such lignocellulosic bioenergy biorefineries.

### Farmers, Foresters and Mariners Becoming an Integrated Part of New Bio-Based Value Chains

The bioeconomy starts with biomass producers. Biomass in its broadest definition comes from forestry, marine, agricultural resources, as well as from its industrial processing, and biological wastes arising from various sectors of society. Farmers, foresters and mariners are critical and essential stakeholders in the bioeconomy yet much of the initial development of the bioeconomy took place without their input. The bioeconomy will not be socially sustainable without buy in and benefit to these stakeholders. Farmers that are organized into Cooperatives that extend their business from farm to market through food processing enterprises have a competitive advantage with regards to moving into the bio-based industrial sector. They can inherently access and inform the entire value chain for feedback loop exchange of knowledge from primary production to processing and from processing to primary production. By contributing to refining and optimizing the sustainable production of biomass, suitable for upgrading to higher value products, farmers earn the license to be valued partners in the value chain. They can make multiple contributions to a sustainable circular bioeconomy through innovation of agricultural practices, e.g., water conservation, reduction or avoidance of pesticides, reducing GHG emissions, strengthening resilience through biodiversity, and improving resource efficiency.

### Need for Land Use Change, Freeing Land for Food Production, Forest and Biodiversity Habitats

Currently the production of animal feed takes up almost three quarters of all arable land in EU. Globally the land needed for growing animal feed is approx. 77% of the global arable land^1^ (Ritchie, 2019). Several types of bio-based products can positively impact land use, by making animal feed based on upgrade of industrial side-streams, thereby supplementing animal feed without the need for growing new crops. Furthermore, the reuse of materials can reduce the burden on land use and free up land for food production, forestry and dedicated, non-cultivated land to enhance biodiversity. The aquatic sector can also play a critical role in reducing burden on land, by using marine and freshwater space instead, but also because some aquatic biomass production, like algae aquaculture, not only takes only very limited land space but also has a positive impact on carbon sinking and uptake, inverting the carbon cycle from ocean to land. This plays a crucial role not only in restoring marine ecosystems biodiversity and services capacity, but also in supplying alternative feedstock value chains and creating new social/jobs developments.

### Climate Change and the UN SDGs

Bio-based upgrade of sustainably sourced biomass delivers both to climate change mitigation and to fulfilling a broad spectrum of the UN SDGs (Philippidis et al., 2018b; Heimann, 2019; M’barek et al., 2019). More specifically, efficient use of the biological resources can contribute to improved livelihood: feeding a growing population, by making more food and feed from what is now going to waste (SDG #2 Zero Hunger); production of bio-based health-promoting products such as these, that stimulate gut health for improved resilience of man and animal or that reduce the need for antibiotics in animal husbandry (SDG #3 Good Health and Well-being), and open for access to clean water by developing bio-based control of plant diseases and pests to substitute for chemical pesticides (UNSDG #6 Clean Water). Sustainably sourced and, efficient and responsible use of biological resources can deliver to the UN SDGs focusing on protecting the global biosphere; both terrestrial and aquatic (SDG #14, Life Underwater and SDG #15 Life on Land). Furthermore, bio-based economy can deliver to fulfilling the UN SDGs focusing on changing human and industrial behavior and efforts toward more sustainable practices and technologies: Clean Energy (SDG #7), Decent work and Economic growth (SDG #8), Industry Innovation and Infrastructure (SDG #9), Sustainable cities and communities (SDG #11), Responsible Consumption and Production (SDG #12), and Climate Action (SDG #13).

The climate change challenge and the international agreed reduction of CO_2_ is a very strong global driver toward more efficient use of precious biological resources. The Bioeconomy can help to deliver these ambitions. However, the more efficient use of primary production, as well as producing bio-based substitutes for fossil-based chemicals and materials are not directly counted as positive steps toward meeting the Paris Agreement obligations (UN Paris Agreement, 2016). Notably, the lack of integrating strategically relevant developments and achievements of a bio-based society into the climate change mitigation agenda, is still unfortunately only, to a marginal extent, included in the global COP agenda (COP = Conference of the Parties, (Parker and Karlsson, 2018). This calls for urgent action. IPCC took the first step in 2019, recognizing consumer diets to be of importance for climate change (IPCC and FOOD, 2019).

### Improved Framework Conditions

As stated above, EU strategy and directives have been a driver for the development of the bio-based industries and are expected to be an even stronger driver in the future (EU Directive, 2015). A recent prominent example is the compulsory landing requirement of fishery by-catch (European Environment Agency, 2019). This is expected to provide large volumes of feedstock for making new, higher value products from types of fish, now being caught, but either dumped or downgraded, and thus not used to its full potential. A second example is the new EU directive making it compulsory to sort out discarded textiles; meaning *de facto* that combustion and landfill of textiles are no longer an option. Such resource efficiency directives open up new business and climate friendly opportunities.

## Scope and Development of the BBI-JU Program, 2014–2020

**FIGURE 2:**
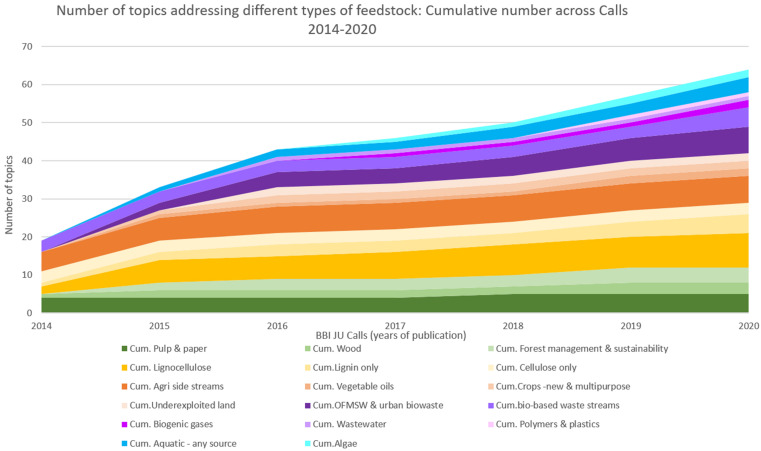
Number of topics addressing different types of feedstock: cumulative numbers across BBI JU Calls 2014–2020.

**FIGURE 3:**
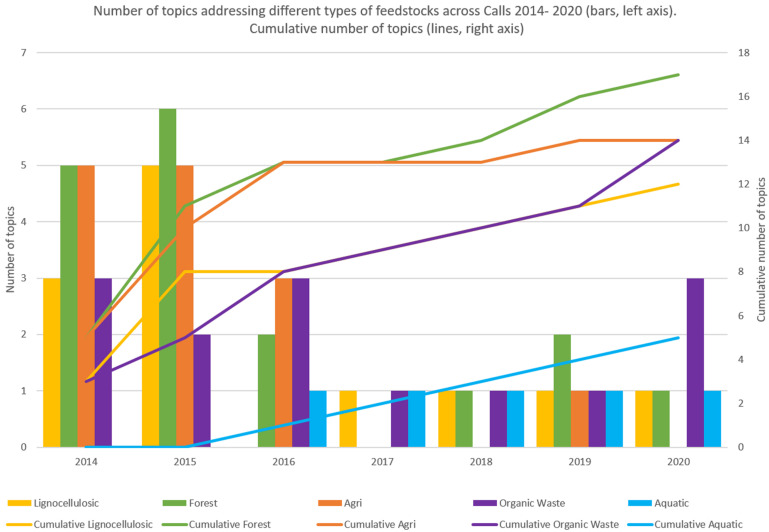
Number of topics addressing different types of feesdtocks across Calls 2014–2020.

The Strategic Innovation and Research Agenda (SIRA 2013 BIC, 2013) was the blueprint for the research and development required to advance the bio-based sector in Europe. It was the basis for the BBI-JU annual work program. The SIRA 2013 identified 5 value chains namely lignocelluosic feedstocks, next generation forestry value chains, agro-based value chains, organic waste, and integrated energy, pulp and chemical biorefineries for the production of advanced transportation fuel, chemicals, materials, food ingredients and feed, and energy. Notably, the feedstock termed “agro-based” covers two very different categories, crop residues and industrial side-streams/by-products. For future analysis, a separation of these two feed-stocks would open for more insightful and informative statistics as upgrading also of industrial side-streams is a significant step forward in developing the bioeconomy. Already in the first BBI-JU call (2014, BBI-JU, 2014) upgrading of a wide range of biomass was included (Figures 2, 3). Processing of the recalcitrant lignocellulosic (yellow) biomass from forestry and agriculture (wood, straw and stover) constituted more than half of the total Call topics. Interestingly, the upgrade of agro-industrial side-streams and wastewater were included already in the first BBI-JU call and a harbinger of future program focus.

The initial processing approach was to make an even more efficient production of a sugar platform, based on which value-added products such as chemicals, materials and fuels could be produced (by microorganisms, living on the sugars; producing the molecules for fuel, chemicals and materials; final product development enabled by integrated use of chemical and biological catalysts). This contributed to establishing a European state-of-the-art industrial knowhow in the area first initiated in the US; and a development beyond this, toward making even higher value products from the recalcitrant biomass.

In a global perspective, the first biorefineries, only focusing on the lowest value products, the biofuels, had to become commercially viable by scaling. In size, in feed stock volume and in capital investment.

The most prominent changes of BBI-JU call topics in the period 2014–2020 was to expand into upgrade of more types of organic solid waste; and to include upgrading of blue biomass; first with emphasis on macroalgae/seaweed; later including also upgrade of fish processing waste and side-streams (Figures 2, 3). Notably, the diagram in these two figures only reflects type of feedstock. It does not depict the rich development of technologies for processing, which took place already in the first calls of BBI-JU. The processing and the product development technologies advanced and diversified significantly, thanks to the efforts of the industries and the consortium partners in the funded BBI-JU portfolio.

### Developing and Diversifying Industry Involvement

**FIGURE 4:**
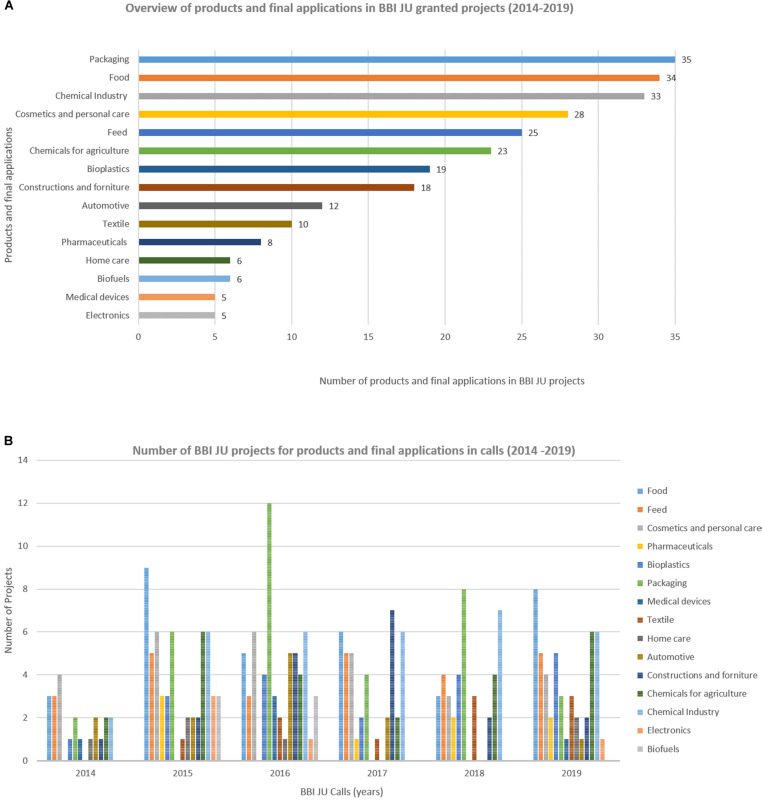
Expanding the portfolio of industries involved in the BioBased Industry Consortium. **(A)** Overview of products and final applications in BBI-JU granted projects (2014–2019). **(B)** Number of BBI JU projects for different products and final applications in calls (2014–2020).

As the BBI-JU became more established, a greater diversity of industries joined the Bio-based industries consortium (BIC) and with that expanded membership the scope of the annual work program expanded (Figure 4).

**FIGURE 5:**
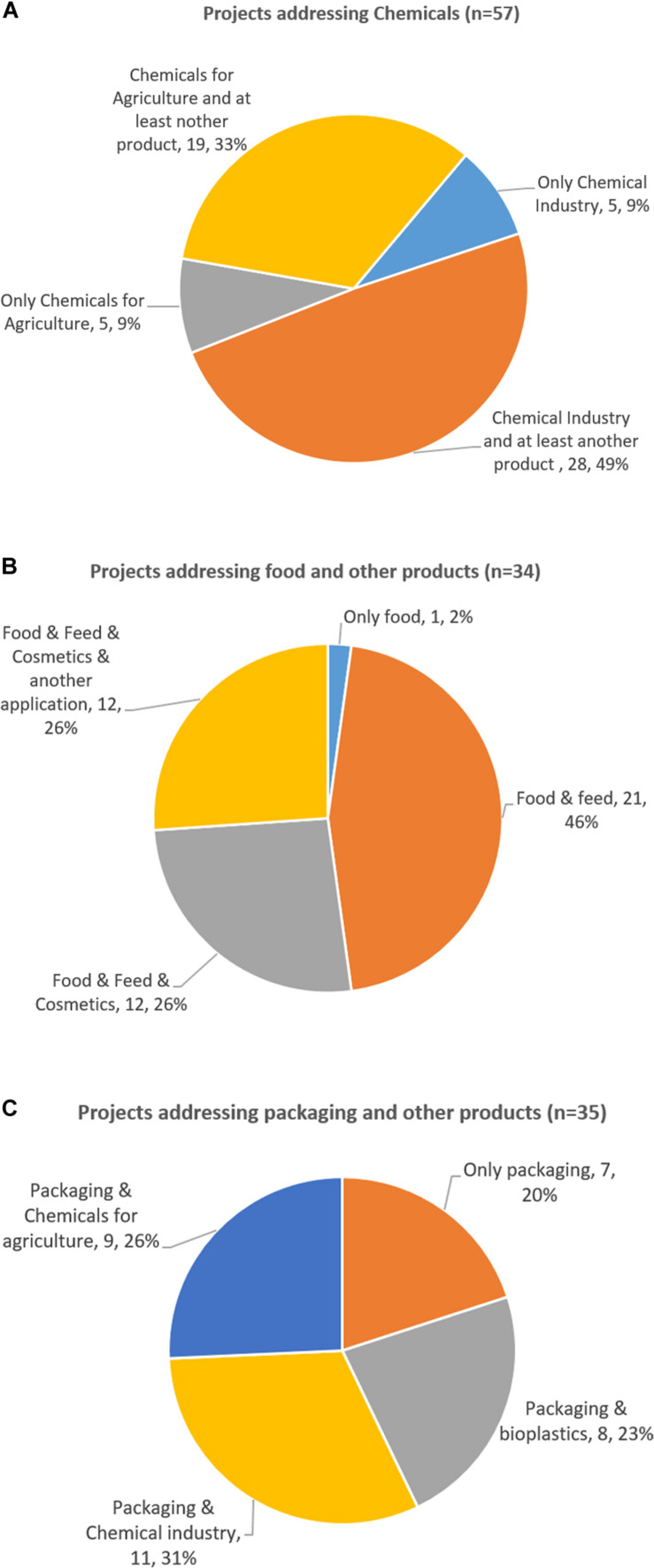
Developing the portfolio of bio-based products. **(A)** Projects addressing biobased chemicals (*n* = 57). **(B)** Projects addressing biobased packaging (*n* = 35). **(C)** Projects for food, feed, cosmetics and health (*n* = 34).

**FIGURE 6:**
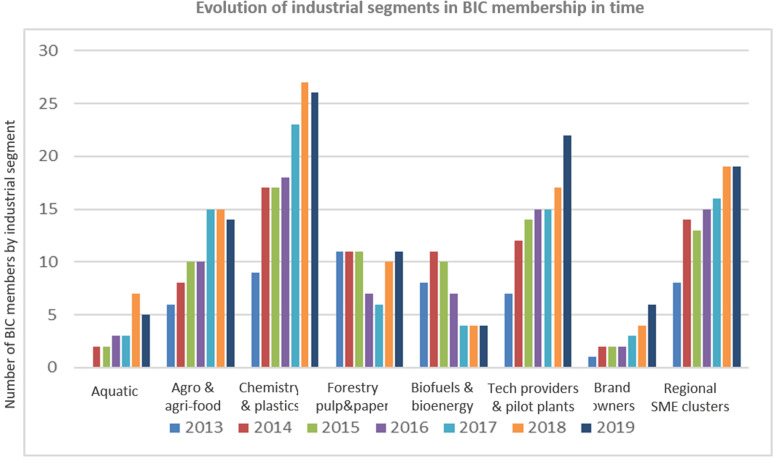
Evolution of industrial segments in BIC membership in time or alternatively Expanding the portfolio of industrial segments involved in BIC.

The broadened and strengthened BIC membership contributed to discussions with the EC, BBI-JU, and its advisory bodies on new priorities, value chains, new products and technologies. From the viewpoint of the Scientific Committee of BBI-JU we observed, that more of our suggestions became relevant and reflected in the topics of the annual work program. For example, an expansion of the target biomass to include residues such as industrial bio-based side-streams, organic wastes, aquatic resources and even bio-based CO_2_ (Figure 3); and a broader spectrum of bio-based products to include products that benefited human and animal health (Figures 5, 6).

Cover the period of 2015–2017, the value chain focus expanded from first focusing on one product, to focusing on multiple products which reflected the ambition of unlocking the full potential of the biomass; by implementing the cascading principle, whereby biomass components, such as proteins are recovered upfront instead of being destroyed in the linear reductionist “sugar platform” processing approach. Higher value products, such as functional food ingredients or new added-value animal feed ingredients could be made from the same biomass that is destined to make chemical building blocks or materials. Circularity and resource efficiency continued to evolve, and the annual work program called for Zero waste biorefineries. Here also a new perspective on the bioeconomy and its role on climate change emerged. While the capture of carbon through the growth of plant biomass and the reduction of GHG emissions from biorefineries through process improvements was well established, the role of biorefineries to capture and use CO_2_ as a resource was not. CO_2_ as a feedstock is now manifest in the Annual Work Plan 2020 call (BBI-JU, 2020a) for demonstration proposals for the use of biogenic CO_2_ as a feedstock for biotechnological and chemical processes. All in all, the market focus and ambitions of the BBI-JU partners, hand in hand with the advice from the two advisory bodies to BBI, the Scientific Committee and the State Representative Group, moved the BBI program forward at impressive speed (Figures 1–6).

## Organizational Highlights

### Developing the BBI-JU Funding Instruments, to Promote Integrated Research Collaboration

**FIGURE 7:**
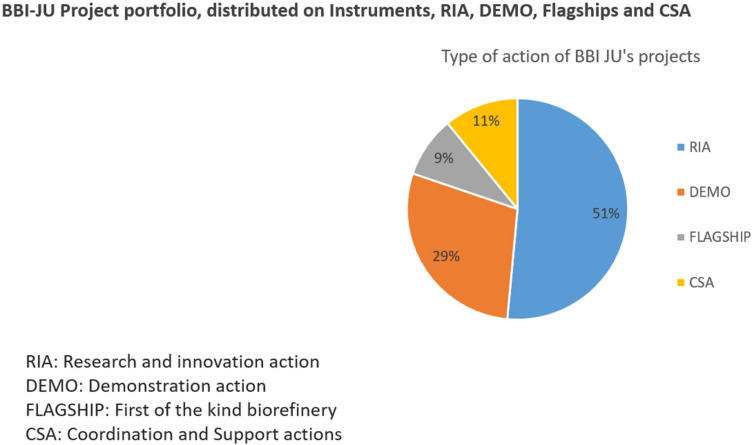
BBI-JU Project portfolio, distributed on Instruments, RIA, DEMO, Flagships and CSA.

Coinciding with the broadening of the scope of the BBI calls, a refinement of the BBI instruments took place. BBI-JU funding instruments span the technology readiness scale i.e., Research and Innovation Actions (RIAs), Innovation and Demonstration actions (DEMOs), Flagship biorefineries (FLAGs) and Coordinated Support Action instruments (CSA), see Figure 7 and link to full description of instruments (BBI-JU, 2020b). Life cycle analysis was essential for DEMOs and FLAGs but life cycle thinking was required for RIAs despite the lower TRL. While RIAs are considered far from the market, the program required applicants to examine the economic viability of the products and processes developed and to include an analysis of the value chain and market for the proposed bio-based products. Innovation and Demonstration actions (**DEMO TRL 6–7**) are required to assess the environmental and economic impacts of the developed processes and/or products on different stakeholders and actors involved in the value chain. All projects, if applicable, are also expected to analyze social impacts, in particular the potential for job retention and/or job creation, and training needs for an appropriately skilled workforce. **Flagship** actions (TRL 8–9) must fully assess the environmental, economic and social impacts of the products or processes developed. **CSA**, Coordinated Support Action gives added-value to the BBI-JU portfolio by researching cross cutting aspects of major importance to the bioeconomy as such as education, stakeholder engagement and inclusion (just transition), empowering SMEs, standards and regulation, financial instruments, and societal impact, which are not possible to capture in a single project.

These four different BBI-JU instruments, RIA, DEMO, FLAG, CSA provided a framework for integrating both large industries and SMEs in the same consortia along with academic research institutes as well as civil society NGO’s. In addition, BBI-JU has been stimulating mobility among talented young scientists across Europe. Similarly, BBI-JU has been instrumental in attracting European as well as international investments in Demo and Flagship projects, de-risking conditions for investors to enter into promising EU bio-based business. The BBI status as Joint Undertaking, with its own board, advisory groups (SC and SRG), secretariat, instruments and industry led modality paved the way for prominent influence and impact on the growth of the bioeconomy in EU as well as development of the here from derived overall societal and climate and environmental benefits.

## Ambitions, Goals and Impact of BBI-JU Flagship Projects

### 2014

FIRST2RUN BBI-JU Flagship aims at demonstrating technology, economic and environmental sustainability at industrial scale of a first-of-kind value chain, valorizing low input, underutilized oil crops; achieved by converting such types of biomass into biobased products, biolubricants, biocosmetics, and bioplastics. By the cascading approach, the residuals from such processes will be valorized for energy, animal feed and added value chemicals. Standardization, certification and dissemination activities will support increased marketability, social acceptability and market penetration.

### 2015

Exilva BBI-JU Flagship will upscale the MFC process from existing pilot plant to full scale flagship plant, viz. from 50 to 1000 tons/year. This requires development of plant organization and quality control as well as establishing appropriate process parameters for a stable full-scale production. By achieving this it is demonstrated that industrial symbiosis between biomass/forest industry and application industries can deliver. The ambition of Exilva is to make MFC commercially available in large quantities for a growing MFC material market.

### 2015

LIGNOFLAG BBI-JU Flagship demonstrates an integrated, value chain-oriented approach to drive bio-based production of both ethanol and chemical building blocks. It builds on combining on-site-enzyme production, tailor-made enzymes, chemical-free pre-treatment, intensive energy integration in combination with new harvesting techniques, smart co-product use, and Life Cycle Analysis (LCA).

### 2015

BIOSKOH BBI Flagship will pave the way for a Second Generation European Circular Bioeconomy by showcasing innovation stepping stones to reach techno-economic viability of lignocellulosic biorefineries. Consortium includes partners of the entire value chain, from feedstock producers, supply chain experts and research partners. BIOSKOH includes dedicated innovation actions for unlocking cascading potential through lignin valorization and 2G bio-chemicals, LCA, Socio-economic impact analyses.

### 2016

AgriChemWhey BBI-JU Flagship will build industrial-scale biorefinery producing integrated symbiotic industrial and agricultural value chains, hereby valorizing annually > 25,000 tons Whey Permeate and De-lactosed Whey Permeate (two major side-streams of dairy processing). Hereby producing lactic acid, polylactic acid, minerals for human nutrition and bio-based fertilizers. Further, AgriChemWhey will establish industrial symbiosis with other local actors for production of sustainable food and feed from other side streams, hereby enhancing circular bioeconomy in agri-food waste. A blueprint of replication plans for other regions across Europe will be developed; hereby contributing to rural growth, competitiveness and job creation.

### 2016

PEFerence BBI-JU Flagship will establish a globally first-of-a-kind, industrial scale (50.000 tons/year), cost-effective FDCA (diacid) biorefinery flagship plant, producing bio-based chemicals and materials (bottles, films, Lego Bricks, polyurethanes) in industrial symbiosis involving also existing facilities. The consortium aims to replace a significant part of fossil-based polyesters, but also technologically superior packaging materials like glass and aluminum with 100% bio-based polyesters (such as PEF). PEF bottles can be recycled and used again as raw material for bottles, as well as for packaging and textiles. During the project, fructose produced via an enzymatic isomerization process from 2nd generation glucose will be assessed. Together with customers and brand owners, 100% bio-based end-products will be demonstrated and validated to ensure fast market deployment.

### 2017

SWEETWOODS BBI-JU Flagship will demonstrate industrial level successful and profitable production of high purity lignin as well as new C5 and C6 carbohydrates from hardwood. SWEETWOODS plant utilizes, based on novel enzymatic solutions all the fractions of the biomass feedstock so efficiently, that min. 95% of its initial carbon content is utilized. From its feedstocks, SWEETWOODS will produce both elastomer foams and high purity sugars for multiple purposes of several industrial market segments. The environmental and socio-economic performance of the SWEETWOODS, plant operation and products are evaluated both by LCSA and commercial viability.

### 2018

FARMYNG BBI-JU Flagship provides an innovative solution for the predicted shortage of protein, through insect production and value-added processing. FARMYNG has as ambition, first of its kind, to develop in industrial scale, automated breeding and processing of insect biomass for the production of value-added animal nutrition. The plant developed for FARMYNG will use the efficiency of mealworm physiology to convert plant biomass to mealworm biomass and transform those mealworms into sustainable proteins and lipids for fish feed and pet food end markets. Insect-derived proteins and lipids results in lower emissions, reduced consumption of water and land. Then goal of FARMYNG is to transfer technology from demo plant to industrial plant, producing 1.500 tons of insect protein and 400 tons of insect oil per month, reaching a productions rate never demonstrated before for insect’s proteins production plant.

### 2018

PLENITUDE BBI-JU Flagship will build a bio-based value chain based around a unique, highly efficient zero-waste process that produces sustainable protein by integrating an aerobic fermentation plant with a conventional first generation biorefinery; hereby producing food-grade fungal protein, based on cereal feedstocks, using an integrated biorefinery setup. Thus, PLENITUDE enables food-grade mycoprotein production (by fermentation of natural, non-GMO filamentous fungus) within an integrated bioethanol refinery. PLENITUDE consortium aims to extend capacity to produce in all, cumulatively 1M tons of protein by 2030, hereby providing replacement for meat from livestock, equal to a reduction of > 5M tons of carbon emissions.

### 2019

ReSolute BBI-JU Flagship aims to pave the way for the production, at full industrial scale, of novel, non-toxic and high performing solvent from wood biomass; hereby addressing the chemical sector challenge for renewable and less toxic chemicals and the need for pulp and paper industry to diversify into new areas. In response to these needs, ReSolute will upscale a proprietary process to produce novel solvents, specialty polymers, flavors and fragrances, pharmaceutical and agrochemical actives. Derivatives of the first commercial product, Cyrene, aims to outperform traditional dipolar aprotic solvents, the latter currently on regulatory pressure due to toxicity. ReSolute brings together 11 key actors along the entire value chain, aiming at enabling its key objective, producing 1,000 metric tons Cyrene based on saw dust.

### 2019

AFTER-BIOCHEM BBI-JU Flagship aims to create multiple new value chains, from non-food biomass feedstocks, by combining anaerobic batch fermentation and esterification. Feedstock may be byproducts of sugar production, such as beet pulp and molasses. In the fermentation process robust mixes of naturally occurring micro-organisms will produce selection of organic acids as well as converting the process residues into a mineral-rich fertilizer. Esterification will convert acetic acid into ethyl acetate and propionic acid into ethyl propionate to maximize product value and minimize waste and energy use. AFTER-BIOCHEM products represent valuable renewable, bio-based, domestically sourced alternatives to petrochemical products, hereby contributing to the EU Action plan for the Circular Biobased Economy.

## Discussion

### Next Steps and New Opportunities

Efforts toward climate change mitigation are often seen as expenditures; needed but costly. However, bioeconomy (making improved use of the raw materials to create value added products, reducing waste, using CO_2_ as a feedstock) can be driven by business. Making more value from the same feedstock strengthens competitiveness and profitability. Furthermore, and most importantly, this approach can drive the environmental impact and contribute to meeting the UN SDG’s and the EU Green deal (European Commission, 2019c). Hereby seeking to deliver climate change mitigation and adaptation as well as providing products, which promote health and welfare for both man, animal and planet, which again provides a strong basis for industrial competitiveness of today and tomorrow. Indeed, improved human and animal health is a rapidly growing area for the bioeconomy where bio-based products can improve gut health and reduce antibiotic use, thus reducing the risk of antibiotic resistance threatening both human and animal healthPlanetary and human health is bolstered by new biomass feedstock types and value chains, like those based on algae culture for example, that not only produce the desired starting biomass material but also positively impact ecosystem and the carbon cycle, allowing a true decarbonization of our economy to occur. Furthermore, reducing or eliminating pesticide and fertilizer use and thus reducing residues in drinking water. In more recent times, and again reflected in the most recent EU Biodiversity Strategy (Europe Central Asia, 2020), Biodiversity is seen as a pillar of resilience in the bioeconomy and the environment and for society-at-large.

The initial direct comparison of a bio-refinery with an oil-refinery may have led the world astray regarding what a biorefinery is. Oil resources are found only in rather few places around the globe, while biomass resources are found all over, from the Arctic to the Tropics. Oil biorefineries are huge installments, requiring huge investments. Biorefineries, which focus on higher value products, can be profitable also in small scale and with lower requirements for investments. More small scale and local biorefineries can stimulate job creation, livelihood and economic development in rural and coastal areas which struggle in a modern fossil-based economy. Clusters of SMEs and start-ups can develop around biorefineries where local engagement and collaborations can be nurtured to build new value chains, integration with primary production (agriculture, forestry and fishery) and generate innovative poles to foster both regional and local development. The local development can be kick-started by identifying underexploited biomass feedstock and the opportunity it represents can be used as a springboard for talented local or recruited entrepreneurs, SMEs and Start-Ups. This development can be stimulated by conducive infrastructures, provided by the municipality; and strengthened by attraction of external public funding, lowering the risk, opening for attracting investors for upscaling the promising value chain operations. The upstart of an EU-wide network of such local, bio-based feed-stock valorization operations, across Europe could inspire and assist more such endeavors to take off not only in the EU but globally.

For decades, global manufacturing has been undergoing outsourcing-derived geographical localization, with an overall trend of moving away from Europe and the USA. Bio-based production has a significant role to play in re-establishing EU based manufacturing industries. This opportunity is further enhanced by the European commission’s drive toward a circular economy where efficiency in materials flows and circular upgrading fit perfectly with the valorization of bio-based resources (including wastes). Recirculated materials (e.g., textiles, materials and chemicals) provide a local resource of new materials, available and accessible as feedstock for new manufacturing creating new business, local development and new jobs. For the first time in decades new growth opportunities are emerging for generating local manufacturing business and jobs, using the re-cycled materials (plastic and textiles) for making new also end-user products.

The impact assessment of BBI-JU activities 2014–2020 reported that an EU industry-driven RTDI program provided momentum in using new knowledge faster. It also provided stronger focus on solving obstacles for upscaling, commercialization and regulatory approval. In the future, one could envisage Demos and Flagship projects to integrate lower TRL components as a means to improve the technologies as biorefineries needs lower TRL research to, make processing more efficient and the quality of the products even higher. Furthermore, the meaningful engagement of primary producers to an industry-focused program is truly pioneering but more can be done here so that the primary producer is not seen solely as a biomass supplier but a partner that can both contribute to and benefit from wealth creation higher up the value chain. The bioeconomy in Europe and globally will benefit significantly by integrating the feedstock producers and providers as key value chain partners. The BBI-JU, keeping in line with Horizon 2020 mission and objectives (European Commission, 2020c), is an international program with partners from outside Europe as part of funded projects, building on this and establishing value-added international collaborations, will deliver win/win solutions: building skills and technologies, generating investments and growing markets, building globally compatible systems. Related to this is a need and necessity for strengthening the European research-support infrastructures, e.g., the biomass conversion relevant and well-curated enzyme databases, Fungal and Bacterial Culture collections, as well as genome and microbiome support systems for sequencing and annotation, including advanced bioinformatics analysis of taxonomy and function. EU investment in this area are, in an international perspective, magnitudes too low. Both the EU interests and the global interests in improved use of biological resources would benefit significantly from stronger EU efforts in bioeconomy. Significant higher EU investments in research support infrastructures represents the shortest and most cost-effective route for strengthening the development of the EU bioeconomy. A strong foundation for Africa-EU partnership was built during the last years of Horizon2020, with a special focus on food security and sustainable agriculture [FNSSA approach (Food and Nutrition Security and Sustainable Agriculture)] (European Commission, 2019a). Since then, the Africa-EU collaboration has strengthened by developing a dedicated Africa-EU Strategy (European Commission, 2020d). It is highly interesting to note that extending the Africa-EU collaboration to include development of the African bio-based economy (upgrading of residues, industrial side-streams and wastes to higher value products) provides an exemplary match to the priorities of the new strategy. An Africa-EU partnership for development of the African bio-based economy has the potential of delivering to several of the priorities of the new Africa-EU Strategy (e.g., green transition, sustainable growth and jobs, as well as collaborative progress regarding migration and mobility).

### Developing the Circular Biobased Economy -the Global Perspective

In a globalized world it is important to place the described EU development of the bioeconomy also in a global perspective. A short review is presented here.

The major global players have chosen different avenues in developing the bioeconomy. The United States (not the least through efforts of DOE, NREL and private companies) had a pioneering start, focusing on breakthroughs in developing the technologies for converting biomass to bioethanol. A favorable investment climate in the United States led to scale up of both United States and EU developed biomass to bioenergy conversion technologies on American soil. However, not all investments were successful, due to low global prices on bioethanol, narrow profit margin from biomass to bulk biofuels, as well as slower development of consumer demand for biofuels for private cars despite Government biofuel blend-in strategies in both the United States and EU. However, United States (and Canada) also took other significant steps toward upgraded use of biomass. Highest priority after bioenergy and biofuels was the production of biobased chemical building blocks and biobased materials as substitutes for fossil-based equivalents. Another significant trend in the United States bioeconomy was to broaden the biomass feedstock from crop and crop residues to also including waste. Civil society in United States took steps to reduce food waste and start-up companies in United States were pioneering in making non-animal based “meat”. The EU pioneered trend of cascading use, unlocking the full potential of a broad selection of feedstocks, residues, side-streams and wastes to higher value products has so far not been implemented in United States.

The development of the Bioeconomy in China has been both fast and efficient in the scaling up of production of bioenergy and biofuels from biomass. Chinese scientists have taken great interest in exploring technologies for producing higher value products from their domestic (and imported) biomass. However, the last Chinese five-year plan gave specific priority to producing energy from biomass to reduce the need for the import of energy and to have supplementation of the fuel market with domestic biofuels. The new five-year plan seems to open up possibilities for production of higher value products from biomass. More food and feed are of crucial importance in a country, which has a very low ratio of arable land to human population. Here it is interesting to note the pioneering Chinese efforts in upscaling production and commercialization of gut-health promoting food products and ingredients (dietary fibers from cereals and sweet potatoes); starting a new chapter of the Chinese traditional health-promoting diet and medicine. COVID-19 has very recently modified and expanded the Chinese overall approach to the bioeconomy. Strong R&D health-related efforts in China are committed to deliver products, which add and improve public health in China. Notably, expressing at the Global Bioeconomy Summit in November 2020 that improved public health is a priority (also) for stimulating the Chinese economy.

The two large global players in agricultural production, Brazil and India both have a wealth of well-educated researchers and engineers with expertise relevant for development of their national bioeconomy. A development which could result in improved use of their vast biological resources. It is however a potential not yet fully exploited. Brazil was the pioneer of the world in producing bioethanol from sugar-cane. Also, technologies for upgrading the residual, lignocellulose-rich residual resource from sugar production, the bagasse has been developed. However, most of the new public biomass conversion R&D in Brazil are within the white biotech area, focusing on substituting chemical processing with bio-processing. India has a strong tradition for resource efficient agriculture e.g., by refining the use of green manure as soil improvement products. Also, the upscaled commercialized production of industrial enzymes in India is remarkable (e.g., the development of enzymes for upgrading leather, substituting for toxic chemicals still used in most of the world). Indian industrial scientists and engineers have made break-throughs in the production of collesterol-regulating statins, through filamentous fungus biotechnology. Within the next one to two decades both India and Brazil have the potential to emerge to be among the leading global players, in producing higher value products from biological resources which are currently wasted or downgraded to lower value products. The number of skilled engineers combined with the volume of available biomass feedstocks in both India and Brazil present a huge asset for future development.

## Conclusion and Recommendations

The EU has made huge transformative progress to its economic model, through the BBI-JU program, mobilizing actors across sectors to collaborate in a new bioeconomy paradigm, which we believe has been made possible by its status as a Joint Undertaking. The BBI-JU program has developed biomass conversion technologies for all types, plant-, animal- and microbial-derived biomass. Technologies applicable for both terrestrial and aquatic biomasses, as well as for industrial side-streams, byproducts and wastes. A broad spectrum of new, bio-based value chains, spanning from simple fossil-substitutes to bio–based products with novel functionalities without a fossil equivalent. Further and most importantly, the BBI-JU program has developed a rich portfolio of food, feed and health promoting products.

Preconditions for supporting the development of Sustainable and Circular Bio-Based Economy in EU are

1. Continue developing and supporting public private partnerships value chain consortia
2. Improve approaches to efficient resource utilization, biodiversity, GHG emissions reduction, production of products for human, animal, biodiversity and planetary health
3. Support that flagships and Demos build will deliver resilience
4. Support a broadened portfolio of small and large biorefineries across Europe
5. Continue the BBI-JU endeavor, by strengthening RTDI for Circular Bio-based Economy
6. Develop the regulatory framework alongside technology validation to monitor and address gaps and obstacles that slow down new knowledge from being used quickly
7. Strengthen knowledge dissemination, capacity building and mechanisms for broadening the implementation of the Circular Bio-based Economy in EU
8. Develop strategy for strengthened international win/win collaboration within circular bio-based economy

Among the gaps and obstacles that slow down new knowledge, to be used quickly also lies the problem of capital allocation, and the often missing, documentation of that the risk-adjusted return of investment, also in the bio-based sector is competitive. Un less signals to the capital market are provided, that in a foreseeable future the risk-adjusted return is comparable to other investments, adequate fund allocation to this very diverse sector might be limited.

In short, continuing this endeavor of public private partnerships consortia, to address societal challenges of importance for both climate change mitigation and feeding a growing global population is needed. The Corona 2020 pandemic taught us that robustness of public health is important for future resilience. But it also came to bluntly remind us that no human prosperity can occur at the cost of the planet’s own health and we desperately need to change the way we use the resources, do business and develop our societies. The robustness of our approach to resource utilization, biodiversity, GHG emissions reduction, production of products for human, animal and planetary health are important for our future resilience. Continuing the BBI-JU beyond its current remit of 2024 is critical to ensure that the momentum built over the last 6 years is not lost and that the Bioeconomy in Europe can grow, evolve and be a leader in climate change mitigation and adaptation as well as in improved and responsible use of the biological resources, unlocking their full potential. The development of the program so that Flagships and Demos deliver research results that will deliver resilience, support a broadened portfolio of small and large biorefineries across Europe, based on environmentally benign processing and sustainably sourced biomass can support the creation of sustainable businesses and communities.

The development of the regulatory framework must be carefully considered alongside technology validation to monitor and further address gaps and obstacles that slow down new knowledge be used quickly and efficiently. Last, but not the least, BBI-JU could be part of a strategic action to proactively strengthen international collaboration – sharing knowledge, technologies and best practices for a circular bio-based society; contributing significantly to meeting the UN development goals, climate change mitigation and adaptation. In a world facing an era of climate change-challenged agricultural production, it is essential to efficiently spread and implement how all the product harvested can be used; instead of the current status where one third or even half is wasted. Significant growth, value and livelihood can be gained by extending the Africa-EU Alliance, going beyond the FNSSA approach (Food and Nutrition Security and Sustainable Agriculture), hereby covering also the value adding bio-based area. Upstart of the new Horizon Europe is an excellent time for initiating this win/win opportunity.

The efficient sharing of pre-competitive knowledge is a way in which the impact of BBI-JU can be broadened even further. Knowledge sharing of the large, non-proprietary biorefinery-relevant platform of technologies already available in the public domain, can stimulate entrepreneurship across all parts of Europe –as well as globally. With new approaches to knowledge sharing, the vision of an efficient, participatory “copy and paste” mechanisms, sharing the non-proprietary part, could become a reality. Such developments are necessary to move quickly ahead in the responsible use of the biological resources in Europe and globally; creating societal, environmental *and* business value at the same time.

## Author Contributions

LL and KC conceptualized the review. LL and KC wrote the manuscript, together with BBI-JU Scientific Committee members. The BBI-JU Management and Secretariat provided BBI-JU project portfolio statistics, feedstock and product overview, and details of development of organizational structure and BBI-JU funding instruments. All authors contributed to the article and approved the submitted version.

## Conflict of Interest

UB-J is the CEO and founder of the companyGate2Growth. Lene Lange is the founder and owner of the company BioEconomy, Research & Advisory. The remaining authors declare that the research was conducted in the absence of any commercial or financial relationships that could be construed as a potential conflict of interest.

## Footnotes

1 https://ourworldindata.org/global-land-for-agriculture
